# Online Learners’ Reading Ability Detection Based on Eye-Tracking Sensors

**DOI:** 10.3390/s16091457

**Published:** 2016-09-10

**Authors:** Zehui Zhan, Lei Zhang, Hu Mei, Patrick S. W. Fong

**Affiliations:** 1 Center of Educational Information Technology, South China Normal University, Guangzhou 510631, China; 2 College of Communication Engineering, Chongqing University, Chongqing 400044, China; 3 School of Economics & Management, South China Normal University, Guangzhou 510006, China; 4 Department of Building & Real Estate, The Hong Kong Polytechnic University, Hong Kong 999077, China

**Keywords:** eye-tracking sensors, online learner, reading ability detection, computational model

## Abstract

The detection of university online learners’ reading ability is generally problematic and time-consuming. Thus the eye-tracking sensors have been employed in this study, to record temporal and spatial human eye movements. Learners’ pupils, blinks, fixation, saccade, and regression are recognized as primary indicators for detecting reading abilities. A computational model is established according to the empirical eye-tracking data, and applying the multi-feature regularization machine learning mechanism based on a Low-rank Constraint. The model presents good generalization ability with an error of only 4.9% when randomly running 100 times. It has obvious advantages in saving time and improving precision, with only 20 min of testing required for prediction of an individual learner’s reading ability.

## 1. Introduction

Reading ability is one of the most important predictors of students’ success in online courses [1]. However, its detection is always complicated and problematic. The traditional way to test students’ reading abilities is to provide different kinds of reading materials and ask students to answer sets of questions according to what they have read. Their reading ability appraisal can then be assessed based on the scores obtained by students. 

However, there are problems with this kind of assessment method. First, the assessment is very time-consuming, normally requiring more than two hours for some simple conclusions. Thus, most of the time, instructors and students do not want to spend time and effort taking these assessments. Second, the results of the current reading ability assessment are not stable, and can be affected by readers’ emotional status and level of fatigue. Third, reading assessment results are sometimes content-related. For example, if students are interested in or have prior knowledge of the reading materials, it might be easier for them to get the right answers in the test. Thus bias might exist if students’ reading ability is judged purely based on their success rate on a reading test, and the results might not be valid and reliable.

To solve these problems, eye-tracking sensors are gradually attracting some educational experts’ attention. Our eyes are the portals of our minds. For an ordinary human being, about 80 to 90% of the information is obtained by our eyes. In reading, readers’ cognitive process is to a large extent dependent on their visual system [2]. Eye-tracking sensors can record temporal and spatial human eye movements [3], which are a natural information source for proactive systems that analyze user behavior, where the goal is to infer implicit relevance feedback from eye tracking indicators (e.g., eye tracking sensors collect information about the location and duration of an eye fixation within a specific area on a computer monitor) [4,5]. Among all kinds of eye movements, pupil diameter, blink rate, fixation rate, fixation duration, saccade rate, saccade amplitude, saccade duration, regression rate and regression path length are the most reported data in eye-tracking sensors, and can be recognized as primary indicators playing critical roles in identifying learners’ reading abilities [6,7]. From previous studies, the function of major eye-tracking indicators is summarized below.

## 2. Major Eye-Tracking Indicators

### 2.1. Pupil

**Figure 1:**
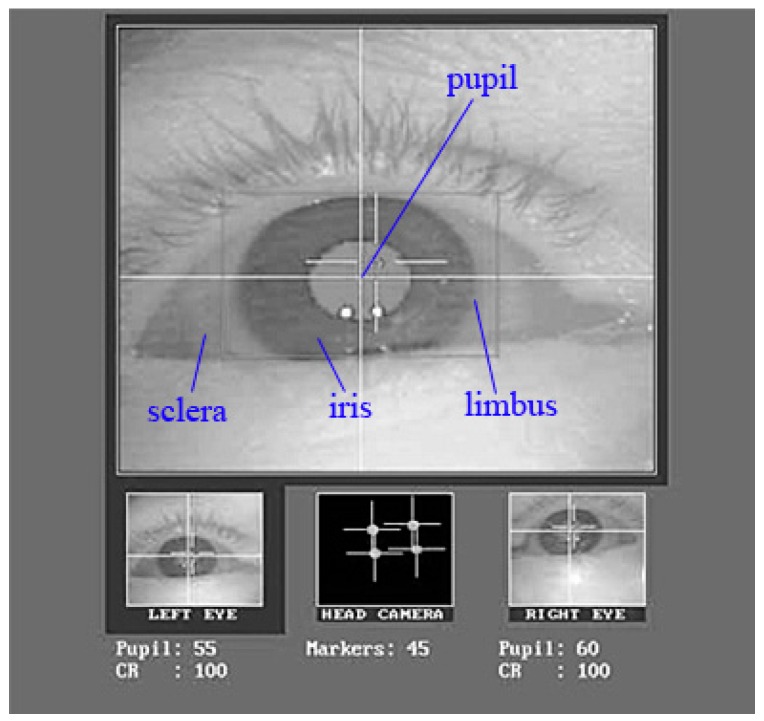
The composition of the eye. Note: The pupil is the area located in the center of the iris. The limbus is the boundary of iris, which is surrounded by the sclera.

The pupil is a hole located in the center of the iris of the eye that allows light to strike the retina [8] (Figure 1). Pupil diameter mainly reflects learners’ degrees of fatigue and interest in a particular learning content [9,10]. Pupil diameter values are typically given in pixels of the eye camera. Van Gerven et al. found that mean pupil diameter is a useful event-related measure of cognitive load in research on education and learning, especially for young adults [11].

### 2.2. Blink

A major function of blinking is to keep the eyeball moist [12]. Blink rate is defined as the number of blinks per second in this research. Several studies have associated the rate of blinks with mental workload [13]. People who need to conduct multiple simultaneous tasks will increase their blink rates more than those who are conducting a single task [14].

### 2.3. Fixation

Fixation indicates the state when the eye remains still over a period of time, such as when the eye temporarily stops at a word during reading. It lasts from some tens of milliseconds up to several seconds. It is generally considered that when we measure a fixation, we also measure attention to that position [15]. Fixation count, fixation rate, and fixation duration are all general fixation-related events.

Fixation count is the number of fixations in the area of interest. It is one of the most used metrics in usability research, scene perception research, and different forms in reading research [16]. 

Fixation rate is the number of fixations divided by a period such as the duration of a trial in seconds, with a general scale of fixations per second. It is reported by Steichen et al. to decline with time on task [17], and was found to be negatively correlated to task difficulty by Nakayama et al. [18]. Fixation duration is the duration of the entire process of fixation. Generally speaking, a longer fixation duration is often associated with deeper and more effortful cognitive processing.

### 2.4. Saccade

Saccade is the rapid motion of the eyes from one fixation to another (e.g., from word to word in reading). Saccades are very fast—typically taking 30–80 ms to complete, and it is considered safe to say that we are blind during most saccades. 

Saccade rate decreases when task difficulty or mental workload increases [18]. Also, saccade rate is increased by arousal and reduced by a higher fatigue level [19,20]. 

Saccade duration is defined as the time a saccade takes to move between two fixations or instances of smooth pursuit [4]. Saccade duration has largely been thought of as a period with no visual intake [21]. Task difficulty has been found to increase saccade duration [14].

Saccade amplitude is the distance travelled by a saccade from onset to offset. The unit is typically given in visual degrees. During reading, saccade amplitudes are limited in length by visual span width, which is around two degrees in average reading situations [22].

### 2.5. Regression

Regression refers to events that move in the opposite direction to the text. It is defined in reading events as going back to the text and re-reading. The regression scan path ends when the point of departure from forward reading is passed and the participant resumes reading left-to-right. The regressions inside words are thought to reflect lexical activation processes (understanding the word), while regressions between words reflect sentence integration processes (understanding how several words relate) [23]. 

Regression rate is counted as the number of regressions per second. Beymer et al. found regression rate values of 0.54 s^−1^ for text with a paragraph width of 9 in, while it was only 0.39 s^−1^ for a 4.5 in paragraph width [24]. 

**Figure 2:**
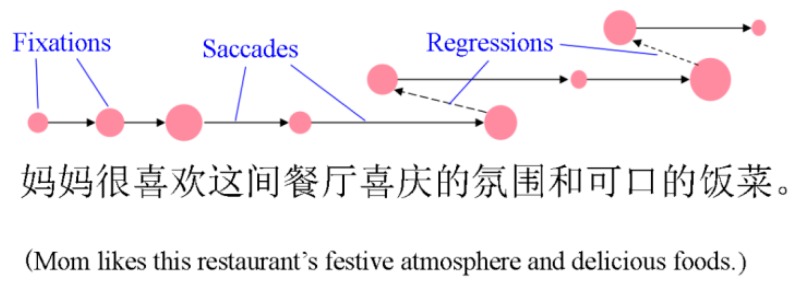
Diagram indicating fixations, saccades and regressions.

Figure 2 demonstrates fixations, saccades, and regressions in text reading. As can be seen, fixations are the red points that indicate that the eyes are concentrating on these positions. The longer the fixation duration is, the bigger the red points are. The saccades are the arrow lines that indicating the fast move between two fixations. The regressions are the dashed arrow lines that indicating the fixation moving backward to re-read the sentence. The original reading materials were presented in Mandarin Chinese and are translated below.

## 3. Research Design

### 3.1. Purpose of the Study

As indicated previously, there is a growing need for effective diagnosis of online learners’ reading abilities in modern distance educational systems. Previous research generally suggested reading tests or questionnaires for assessing reading ability or identifying reading difficulties, but these assessments were either very time-consuming or yielded unstable results, and most of them were limited to English. Eye-tracking might be a quick and effective way to solve this problem. Thus, the purpose of this study was to detect university online learners’ reading abilities by using eye-tracking sensors. That is to say, our main target group is the ordinary university students. However, with the basic results of reading ability detection, those who have reading difficulties could also be identified by specifying the extremely low predicted scores. Therefore, although not serving as the main goal, it may also be potentially used to detect reading disease such as dyslexia. 

### 3.2. Participants

In this study, we collected a total of 6520 freshmen’s Chinese test scores in the National College Entrance Exam and selected 300 participants (the 150 with the highest scores and the other 150 students with the lowest ones) as potential samples. These 300 participants were then invited to our lab to take a Chinese reading assessment test and the Stanford-Binet IQ test. 

The reading assessment test used for the study was created based on previous reading assessment instruments used in University Chinese courses, which contained 58 test items with a maximum score of 100. The internal consistency reliability for the test was determined to be 0.839. A self-reported survey was then served as a way to judge students’ reading abilities. The kappa coefficient was 0.8 (*p* < 0.001), indicating a satisfactory test validity. The reading scores achieved by the participants are ranged from 48.8 to 90.83. 

Finally, 74 students (36 with higher and 38 with lower reading abilities) were identified as target participants for the eye-tracking experiment. They were all freshmen from public universities in south China, aged from 17 to 21 years old. Most of them came from middle class families. All participants had similar IQ level, and were sighted people with normal or corrected vision and no blindness or color blindness. 

### 3.3. Research Steps

To achieve the final goal of reading ability detection, we need to find out sensitive eye-tracking indicators for detection and to build up a computational model to estimate reading abilities based on them. Specifically, the whole study involved the following five steps:

*Step 1*: Eye-tracking indicator screening: According to relevant theories and previous research, pupil, blink, fixation, saccade, and regression data were chosen as the major sets of eye-tracking indicators for building the model.
*Step 2*: Participant screening: A total of 6520 freshmen’s university entrance exam scores were collected, and we chose 300 students as target participants according to their Chinese exam scores, i.e., the 150 with the highest and the 150 with the lowest scores.
*Step 3*: Reading ability assessment and IQ test: Through reading ability assessment, we identified the 36 participants with the highest reading scores and the 38 students with the lowest reading scores. Using IQ tests, we made sure that all participants had a similar IQ level.
*Step 4*: Eye-tracking experiment: An eye-tracking experiment was conducted to collect eye movement data from learners with good or poor reading abilities, and then identified these sensitive eye-tracking indicators to assess learners’ reading abilities. This step of the experiment would be described in detail in Section 4.
*Step 5*: Computational modeling: We built up the computational model with sensitive eye-tracking indicators by using the multi-feature regularization learning mechanism based on Low-rank Constraint. Experimental data was split strategically. Half of the samples were used for model building and the other half for testing the generalization ability of the model. This step of model construction would be described in detail in Section 5.

## 4. Eye-Tracking Experiments

### 4.1. Experiment Setup

**Figure 3:**
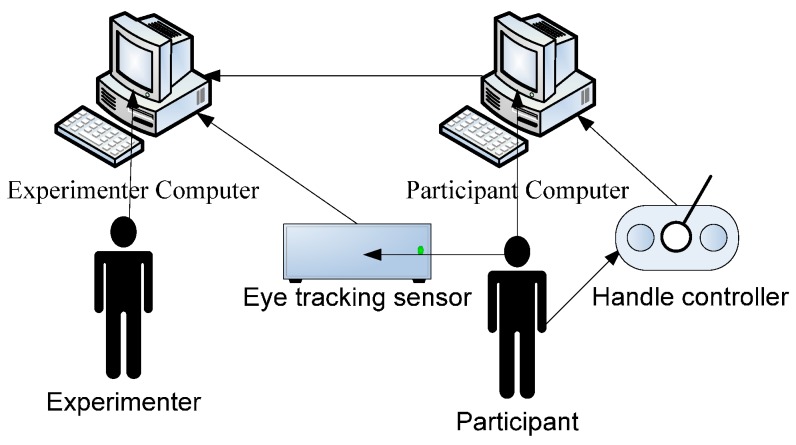
Experiment Setup. Note: The participant’s computer is located in front of the experimenter’s computer, thus the experimenter can observe the participant’s behavior when doing the experiments. The eye-tracking sensor is an Eyelink II helmet that is worn on the participant’s head. A handle controller is placed in front of the participant, for inputting answers to the test.

The experiment took place in the eye-tracking lab. The environmental setup is shown in Figure 3. Two laptop computers were linked together. The experimenter’s computer could control and present the reading materials to the participant via the computer in front of him/her. The participant’s computer was set at a refresh rate of 120 Hz, and the screen resolution was 1024 × 768 pixels. The distance between the participant and the computer was 70 cm. The EyeLink II eye-tracker (SR Research, Ottawa, Ontario, Canada, with a sampling rate of 500 Hz) was placed in front of the participant to collect eye movement data in real time. Participants could use the control handle to reveal the next page of reading materials. Also, they could answer the questions that appeared after the reading materials by pressing certain buttons on the handle.

### 4.2. Experiment Procedures

Seventy four participants were invited to take part in our eye-tracking experiment. Each was assigned an ID and then invited to sit in front of a computer with the experimental program setup. When participants were ready, they would press the blank button and read the guidelines. We made sure that participants fully understood the whole experimental process, and then started to present five trials for practice. Following the practice trials, the real experiment began. 

The experimental trails are all regular sentences relevant to our daily life, which normally contained about 20 characters for each sentence and presented in a single line. All the words were drawn from the Guangming Daily Corpus, and all the sentences were matched with identical sentence structure and balanced sentence length and word frequency.

**Figure 4:**
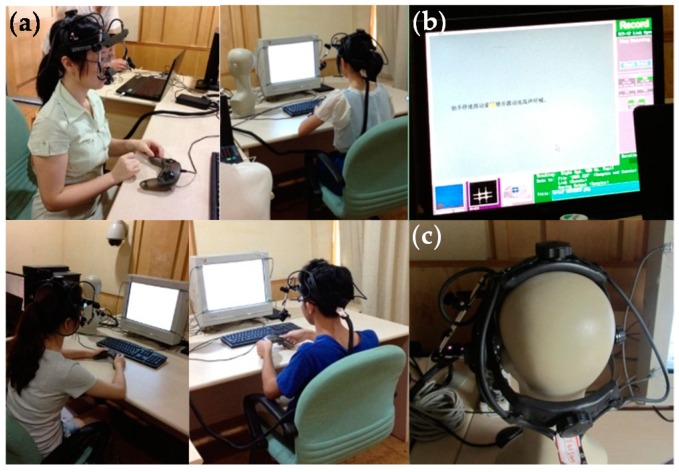
Experimental process: (**a**) Participants doing the eye-tracking experiment in the lab; (**b**) The calibration process of the eye-tracking sensors; (**c**) The eye-tracker helmet (EyeLink II) used in this experiment.

During the experiment, Participants could control their reading speed by pressing the blank button on the keyboard. When they pressed the blank button, the current sentence would disappear, and the related question or the next sentence would appear. The participants could use the control handle to answer the questions based on what they had read. Pressing the left button on the handle indicated “Yes”, and pressing the right button on the handle meant “No”. The whole experiment took about 20 min for each participant. Figure 4 shows the experimental process and equipment used.

## 5. Computational Model and Algorithm

### 5.1. Model Formulation

As illustrated in our experiments, there were 42 trials for each experimenter. Let $X_{i}\in R^{N\times D}$ denote the training data of the *i*-th trial (*I* = 1, …, *m*) and $Y\in R^{N}$ denote the ground-truth reading score vector, where *N* denotes the number of experimenters, *D* denotes the number of indicators, and m denotes the number of trials (*m* = 42 in this paper). Assume that $w_{i}\in R^{D}$ and *b_i_* correspond to the weight coefficient vector and bias for the *i*-th trial, which can be used to approximate the reading scores, then for joint learning of trial *m*; in this paper, we propose a Multi-Trial Joint Learning model (MTJL), which is used to minimize the following objective function, shown as:

(1) $$\begin{array}{l} \underset{w_{i},b_{i}}{\min}\sum_{i=1}^{m}{\left\|X_{i}w_{i}+1_{N}b_{i}-Y\right\|}_{2}^{2}+\lambda \sum_{i=1}^{m}{\left\|w_{i}\right\|}_{2}^{2}+\gamma \cdot rank\left(W\right)\\ \;s.t.\;\lambda ,\;\gamma \geq 0 \end{array}$$

where $W=\left[w_{1},w_{2},\cdots ,w_{m}\right]\in R^{D\times m}$, $1_{N}$ denotes a *N*-dimensional full one vector, and *λ* and *γ* denote the regularization parameters. Both of the regularization constants are equal to 1. In the MTJL model (1), the first term represents the multi-linear prediction, the second term denotes the regularizer of the learned $w_{i}$ for controlling the model complexity, and the third term is a group low-rank constraint on the **W**, which aims to enhance the relatedness of the *m* trial.

Due to the discrete nature of the *rank* function, it becomes difficult to solve Equation (1) directly. Generally, the nuclear norm or trace norm ${\left\|W\right\|}_{*}$ (i.e., the sum of singular values of **W**) is replaced as a good convex surrogate of the rank function rank(W). The ${\left\|W\right\|}_{*}$ can be represented as:

(2) $${\left\|W\right\|}_{*}=Trace\left(W^{T}{\left(WW^{T}\right)}^{-\left(1/2\right)}W\right)$$

where *Trace*(·) represents the trace operator of a matrix and the superscript (·)^T^ denotes the transpose of a vector or matrix.

Therefore, the objective function *J*(**w***_i_*,*b_i_*) of the proposed MTJL model is reformulated as:

(3) $$\begin{array}{l} J\left(w_{i},b_{i}\right)=\sum_{i=1}^{m}{\left\|X_{i}w_{i}+1_{N}b_{i}-Y\right\|}_{2}^{2}+\lambda \sum_{i=1}^{m}{\left\|w_{i}\right\|}_{2}^{2}+\gamma {\left\|W\right\|}_{*}\\ =\sum_{i=1}^{m}{\left\|X_{i}w_{i}+1_{N}b_{i}-Y\right\|}_{2}^{2}+\lambda \sum_{i=1}^{m}{\left\|w_{i}\right\|}_{2}^{2}+\gamma \cdot Trace\left(W^{T}{\left(WW^{T}\right)}^{-\left(1/2\right)}W\right) \end{array}$$

Finally, the proposed MTJL model can be formulated as:

(4) $$\begin{array}{l} \underset{w_{i},b_{i}}{\min}\sum_{i=1}^{m}{\left\|X_{i}w_{i}+1_{N}b_{i}-Y\right\|}_{2}^{2}+\lambda \sum_{i=1}^{m}{\left\|w_{i}\right\|}_{2}^{2}+\gamma {\left\|W\right\|}_{*}\\ \;s.t.\;\lambda ,\;\gamma \geq 0 \end{array}$$

### 5.2. Optimization Algorithm

From the objective Equation (3) of the MTJL, we observe that it is convex with respect to **w***_i_* and *b_i_*, respectively. The close-form solution is easily solved by iteratively computing the partial derivation of the objection Equation (3) with respect to **w***_i_* and *b_i_*, respectively. Therefore, we have:

(5) $$\left(\partial J\left(w_{i},b_{i}\right)\right)/\left(\partial w_{i}\right)=X_{i}^{T}\left(X_{i}w_{i}+1_{N}b_{i}-Y\right)+\lambda w_{i}+\gamma \left(1/2\right){\left(WW^{T}\right)}^{-\left(1/2\right)}w_{i}$$

(6) $$\left(\partial J\left(w_{i},b_{i}\right)\right)/\left(\partial b_{i}\right)=1_{N}^{T}\left(X_{i}w_{i}+1_{N}b_{i}-Y\right)$$

Let $\left(\partial J\left(w_{i},b_{i}\right)\right)/\left(\partial w_{i}\right)=0$ and $\left(\partial J\left(w_{i},b_{i}\right)\right)/\left(\partial b_{i}\right)=0$, then we can get:

(7) $$w_{i}={\left(X_{i}^{T}X_{i}+\lambda I+\gamma D_{r}\right)}^{-1}\left(X_{i}^{T}Y-X_{i}^{T}1_{N}b_{i}\right),\;i=1,\ldots ,m$$

(8) $$b_{i}=\left(1/N\right)\left(1_{N}^{T}Y-1_{N}^{T}X_{i}w_{i}\right),\;i=1,\ldots ,m$$

where:

(9) $$D_{r}=\left(1/2\right){\left(WW^{T}\right)}^{-\left(1/2\right)}$$

The specific algorithm in implementation is summarized as Algorithm 1. After the algorithm, the optimal **w***_i_* and *b_i_* are then used to predict users’ reading ability.

**Algorithm 1.** MTJL for reading ability prediction**Input**: $X=\left[X_{1},\ldots ,X_{m}\right],\;Y,\;\lambda \;\mathrm{and}\;\gamma .\;$
**Output**: **w**_1_, **w**_2_, …, **w***_m_*, **b**.**Procedure**:**Step 1**. Initialize **w***_i_* and *b_i_*, *I* = 1, …, *m*.**Step 2**. For iteration *t*, update $W_{i}^{t}$, $b_{i}^{t}$ and $D_{r}^{t}$
$b_{i}^{t}$ using (7), (8) and (9), respectively.**Step 3**. Compute the objective function value *J*(*t*) using (3).**Step 4**. Check convergence. If |J(t)−J(t−1)|≤ε terminate; else go to step 2, where ε is a very small positive value.

### 5.3. Model Features

The final model has three major features: (1) Each participant undergoes multiple trials in the experiment. This model establishes a novel way of setting up a joint-learning model by using multiple trial data. (2) Models such as the neural network model have a slow optimization process. This model can obtain analytical solutions speedily. (3) Through the study of the constraints of W, this model can achieve better generalization solutions.

## 6. Results

### 6.1. Sensitive Indicators

Thirteen sensitive eye-tracking indicators identifying online learners’ reading abilities were elicited by one way ANOVA and are listed in Table 1.

As can be seen, pupil diameter is a sensitive indicator for detecting good and poor readers. This is not surprising. Previous research has indicated that drowsiness and fatigue decrease pupil diameter [25], while positive emotion and anticipation increase pupil size. When a person is faced with interesting and enjoyable stimuli about which he is more curious, his pupils will enlarge [26]. In addition, pupil size can also reflect a learner’s perception of task difficulty. When task difficulty increases, the amplitude of pupil dilation will become bigger [27]. Thus, from pupil size, we can at least judge learners’ cognitive load, fatigue, and interest during reading, and we can use it to identify their reading abilities.

Blink count and blink rates are both sensitive indicators for identifying learners’ reading ability. As concluded by previous studies, blink rate is a robust measure of fatigue and mental workload [13,14]. Blink rate also reflects the degree of interest and joyfulness. Increased blink rate is associated with negative emotions such as tension and anxiety, while decreased blink rate is often linked to a cheerful psychological state [28]. Online learners with better reading abilities usually have better reading habits to avoid getting tired, and may not find reading tasks as difficult as poor readers, thus blink-related indicators also have the power to identify reading ability.

Fixation count, fixation rate, fixation duration and fixation position X are sensitive indicators, whereas fixation position Y is not. From the perspective of fixation count and fixation rate, a high number of fixations would be indicative of difficulty in interpreting the fixated information or layout [29]. Fixation is also related to participants’ experience. Experts have fewer fixations in their domain of expertise. For example, chess experts have a larger visual span, therefore making fewer fixations than their less experienced counterparts [30]. It is believed that fewer fixations are required to locate objects once they have been encoded and a memory representation accumulated through multiple fixations [31]. In this sense, fixation count and fixation rate should be able to distinguish poor readers from proficient ones. From the perspective of fixation duration, some researchers have argued that expertise in certain fields, such as chess, art, and goalkeeping, results in longer and fewer fixations than found in novices [32,33]. Shorter fixation duration indicates a higher mental workload [34]. With increased skill, more information is extracted around the point of fixation, making eye movements more efficient overall [15]. Thus fixation duration can also be a suitable indicator for identifying reading abilities. From the perspective of fixation position, the x-coordinate is sensitive, but the y-coordinate is not. Learners with better reading ability might be able to understand the whole sentence more promptly, by reading only several words with fewer fixations. This is why the x-coordinate is a sensitive indicator. However, during reading, the y-coordinate is random and will not change much, thus it might not be a suitable indicator for assessment.

Saccade count, saccade rate, saccade duration, and saccade amplitude are all sensitive indicators for testing reading ability. Saccade count and saccade rate can indicate task difficulty, mental workload [18], and readers’ fatigue levels. Saccade duration can indicate task difficulty [35]. A decrease in saccade amplitude would indicate a high level of cognitive load, resulting in “tunnel vision” [36]. Basically, longer saccades are more exploratory, thus poor readers and those with dyslexia might exhibit shorter than average amplitudes. Regression count and regression rate are both sensitive indicators, while regression length is not. This result is consistent with previous studies that have reported that readers with dyslexia have more regressions than normal readers [37,38]. Regression length is not sensitive to reading ability, which means that reading ability does not affect regression path length to a large extent.

### 6.2. Indicator Weights

**Figure 5:**
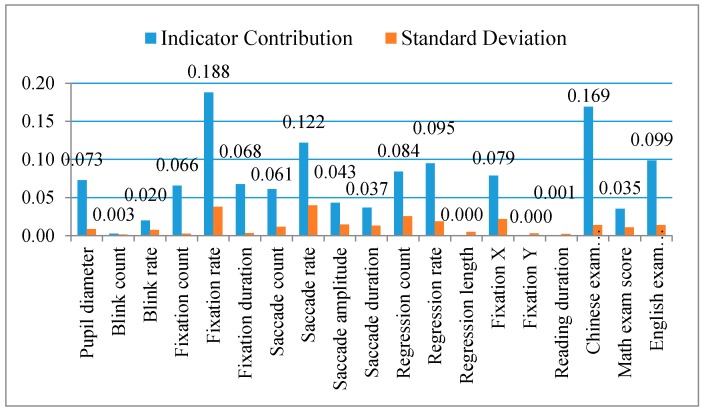
Eye-tracking indicator contribution in the computation model. Notes: As can be seen, fixation rate, saccade duration, and Chinese exam score were the factors that contributed most to the computational model, while blink count, fixation position Y, and reading duration contributed the least to the model. Most of the indicator contribution is consistent with the empirical data, except the blink count and blink rate, which make very little contribution to the model.

During the computational modeling process, we strategically split the 74 students into two halves, and then used 37 of them to train the computer and build up the model. The other half of the sample was used to test the model generalization ability, in order to guarantee the model fit. Figure 5 presents the contributions of the indicators used to detect learners’ reading ability, which would be built into the computational model as indicator weights. All the data has been normalized before building into the MTJL model.

As can be seen in Figure 5, fixation rate and saccade rate make the highest contribution (>0.1) to the model. This is consistent with the results of our empirical study and the previous research indicating that proficient readers make fewer fixations than beginners, because expert readers are capable of faster processing of information conveyed in the form of high-resolution details [39]. This implies that an expert’s fixations are more efficient. Saccade rate can also indicate reader’s mental workload [18], thus good readers usually have a lower saccade rate than poor readers. In this connection, fixation rate and saccade rate both make significant contributions to the model. 

Pupil size, fixation count, fixation duration, saccade count, regression count, regression rate, and fixation position X also make major contributions (>0.05) to the model. This result is also consistent with the empirical data and previous research. It is especially true that both the regression rate and regression count indicators contributed more than 0.08 in this cohort. The poor readers’ regression rate and regression count was much higher than that of good readers, while regression length made almost no contribution to the model. The reason might be that readers make regressions when they feel uncertain, thus the number of regressions that occurred in the sentence area and within a certain period of time is more important information than the length of the regression in the prediction model. No matter how far the regression points travel, once regression occurs, reading uncertainties have been captured. 

Compared to the other four families of indicators, the blink family makes very little contribution to the computation model (<0.03). This is contradictory to previous research as well as our empirical findings, and may be due to the fact that blinking happens at a much lower frequency than the other families of indicators. Not every fixation and trial is accompanied with blinks. Therefore, in the real-time data, the blink indicators have some empty values, which might affect the accuracy of the predicting model. Thus, in the final model, the contribution of the blink-related indicators is limited.

We also found that the “rate” indicators (e.g., fixation rate, saccade rate, blink rate, regression rate, etc.) contributed more than either the “count” indicators (fixation count, saccade count, blink count, regression count, etc.) or the “duration” indicators (fixation duration, saccade duration, etc.). This might imply that “rate” is a more balanced set of indicators, because it contains information regarding both “count” and “duration”.

In addition to the sixteen eye-tracking indicators we examined in this empirical study, we also included the participants’ Chinese, math, and English scores in the university entrance exams in the model in order to predict university students’ reading ability. Since the model can adapt to the actual data and intelligently adjust the indicator weights, in the predicting model for students without university entrance exam scores, alternative data such as General Examination Scores could be used instead. As can be seen in Figure 5, the Chinese exam score contributed the most and the math the least to the model, which are reasonable results and comparable to real-life situations.

To sum up, fixation rate, saccade rate, and the Chinese score in the university entrance exam are the eye-tracking indicators with the greatest contributions (>0.1) to the whole computational model. Fixation duration, pupil size, fixation position X, saccade amplitude, saccade duration, regression count, regression rate, and English exam score are also sensitive indicators with medium contributions (>0.05) for identifying students’ reading abilities. In other words, shorter fixation duration, smaller pupil size, smaller fixation position X, larger saccade amplitude, shorter saccade duration, lower regression count, and smaller regression rate indicate learners with higher reading ability. Blink count, blink rate, saccade amplitude, saccade duration, reading duration, and fixation position Y made the least contribution (<0.05) to the model. In particular, blink count, fixation position Y, and reading duration are indicators that made almost no contribution to the model.

### 6.3. Model Fit and Consistency

**Figure 6:**
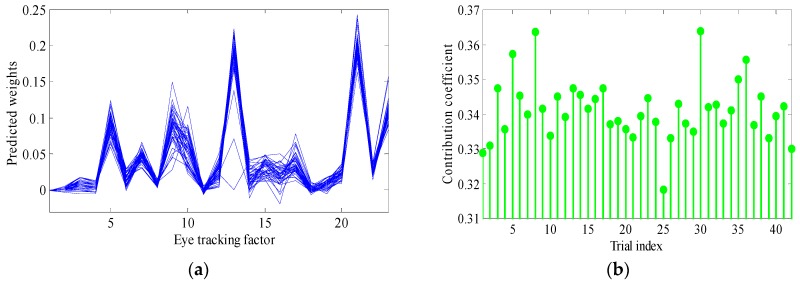
(**a**) Indicator contribution of all 42 trials and (**b**) contribution coefficient of trials.

In order to test and visualize for consistency, the computational model was randomly run 100 times, with an average error of 4.91 out of 100 and a standard deviation of 0.94, indicating a satisfactory fit. The 42 trials of the 74 participants’ data are collated in Figure 6. Figure 6a shows the 72 participants’ data on the contribution to every indicator. Each line represents one participant. As can be seen, the contributions of all the 42 trials do not vary to a large extent. They have a similar trend, thus showing an acceptable model consistency. Figure 6b presents the average contribution of each trial. As can be seen, Trials 8 and 30 had the highest (0.365) and trial 25 the lowest contribution coefficient (0.32). In total, the difference among the 42 trials is about 0.04, which also shows an acceptable consistency of the model.

**Figure 7:**
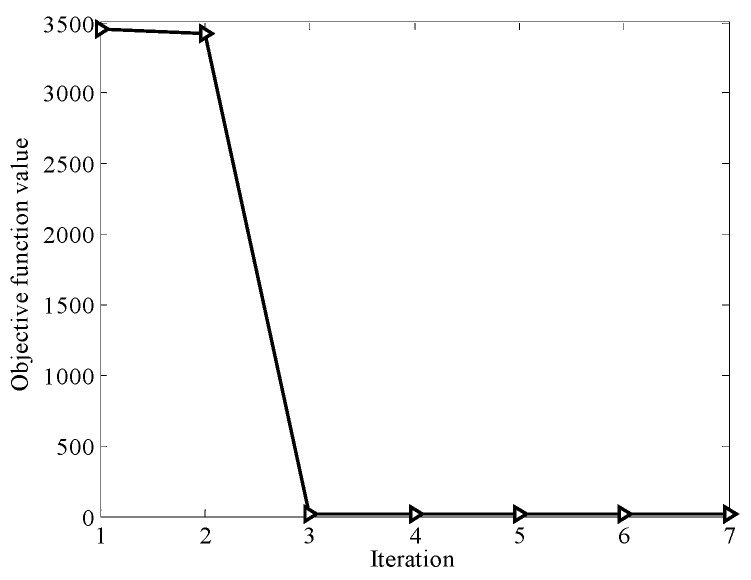
Convergence curve of objective function.

Figure 7 represents the convergence curve of the proposed MTJL model in iterations. We can see that after 3 iterations, the model can converge to a minimum value, which shows that the proposed model is computationally efficient to find the optimal parameters (i.e., W and b) for the reading ability test.

## 7. Discussion

Teaching students in accordance with their aptitudes is the dream of every educator. In this sense, the identification of each student’s ability is crucial in the process of teaching and learning. Reading ability is a very important factor that affects online learning. Thus learners might want to know more about their reading ability before participating in online courses. On the other hand, online educators seldom adjust learning materials according to students’ reading ability. One possible reason might be that testing students’ reading ability is very time-consuming, and also that validity and reliability cannot be guaranteed from a single test. If we can solve this problem and identify learners’ reading ability more accurately and promptly, then online instructors would be able to guide learners in more targeted ways, present knowledge to them in a personalized manner, and ensure the effectiveness of online learning. In order to improve this situation, we are trying to apply eye-tracking technique to learners’ reading ability testing. Since the eye-tracking sensor can record a reader’s eye tracking and other related eye movement parameters, it might be more objective and prompt to identify learners’ reading abilities and habits so as to provide a personalized online learning service. With the help of image-acquiring technology in cameras embedded in mobile equipment (e.g., laptops, mobile phones, etc.), we could also test students unconsciously. 

Therefore, the primary purpose of this paper is to use eye-tracking sensors to detect online learners’ reading abilities. Fourteen eye-tracking indicators (pupil size, blink count, blink rate, fixation count, fixation rate, fixation duration, fixation position X, saccade count, saccade rate, saccade duration, saccade amplitude, regression count, regression rate, and regression length), that were supported by a theoretical background, were identified as sensitive indicators, and have been built into the MTJL model that applying the multi-feature regularization machine learning mechanism based on Low-rank Constraint. From the model indicator weights, we can find that some indicators contribute more than others. Fixation duration, pupil size, fixation position X, saccade amplitude, saccade duration, regression count, and regression rate are the eye-tracking indicators with the highest weights. Thus these are the most sensitive indicators for identifying students’ reading ability. Fixation position Y, regression path length and math scores in university entrance exam are also included in the model, but they make minor contributions to the reading ability prediction. The average error of the model is 4.91 out of 100, and the standard deviation is 0.94. This is about 95% accurate when predicting, which indicates a satisfactory fit for predicting online learners’ reading abilities.

### 7.1. Model Comparison

The current predictive model was evaluated by making comparison with previous predictive assessments. We listed the previous studies in Table 2, and compared them one by one to our model afterwards.

Tomboer et al.’s study tried to develop an objective and powerful method for diagnosing first-year university students with dyslexia. This method used 201 items from questionnaires and 242 items from 10 tests as predictors in two separate analyses with adjusted criterions. This method was able to identify 74 dyslexic and 369 non-dyslexic students. However, among the total sample of 495 students, 10.5 % could not be identified [40]. Compared to our model, this assessment is rather time-consuming and required a lot of effort to complete the questionnaires and tests.

Top et al. tried to establish a predictive model that combined maximum predictive power (in terms of prediction accuracy) with the smallest number of predictors to discriminate dyslexic students from normal readers. They initially selected a total of 27 variables for analysis, and found out the model with three predictors (word reading, word spelling, and phonological awareness) came out as the best. Learners’ predicted probability of being dyslexia can be calculated through a specific equation given the three test scores. The average prediction accuracy on the test data was 90.9% [41]. This assessment has already considered the “time and effort” issue during reading ability testing, while our model would be more time-saving. Although the direct purpose of our model was not to diagnose dyslexia, the predictive results could also help to let instructors pay attention to those whose predictive reading ability is extremely low, which might indicate high probability of being dyslexia.

Wen built up a complete system (three sets) of Chinese reading ability measurement scales applicable to primary schools students. With item analyses, the average item difficulties of the tests was 0.59, and the average item discriminations was 0.32 with significant p value. With the classical measurement theory, the average α coefficient was 0.81. With multi-generalizability theory, the composite absolute error is 5.76% and the generalizability coefficient is 0.81, the index of dependability is 0.80. The correlation between pupils’ test scores and teachers’ grading is 0.71, suggesting high agreement and acceptable criterion-related validity [42]. Compared to this assessment, we have the same assessment purpose of evaluating learners’ reading ability; our model is more time-saving, with smaller predictive error, and with higher criterion-related validity.

Chan et al. assessed school children’s reading ability and discriminate dyslexia by using a 65-item checklist of student reading-related behavioural characteristics that can be observed in the classroom by teachers. Based on a sample of 251 students, the results yielded a correct classification rate of 79% and the kappa coefficient achieved a value of 0.56 (*p* < 0.001), indicating moderately accurate prediction [43]. Compared to the reading test assessments introduced above, Chan et al.’s checklist assessment should be a quick and easier way for assessing reading abilities, however, its correct rate is also bringing down to only 79%.

Tian evaluated school students’ reading ability by a four-perspective structure test, namely recognition ability, comprehension ability, reasonx.xig ability, inducing and summarizing ability. The Cronbach’s *α* is 0.85, which guarantee the test reliability. The correlation coefficient of students’ reading test scores and their Chinese exam score is 0.883 (*p* < 0.01), which showed a good criterion-related validity [44]. Compared to this assessment, although the criterion-related validity of our model is a little lower, we are able save time to a much larger degree.

### 7.2. Model Advantages

According to the model comparison, as can be seen in Table 2, we found that the predictive model in the current study enjoyed relatively high accuracy compared to the existing predictive models. Particularly, there are three obvious advantages to the current model: (1) Saving time: Most of the existing predictive models consist of a reading test or behavior checklist, thus they are very time-consuming. The current computational model is compiled of eye-tracking indicators and university entrance test score, and normally requires about 25 min to complete the reading ability prediction, thus it is a very time-saving method compared to the others; (2) Precision: Most of the previous predictive models aim to classify readers, such as good readers, poor readers, or dyslexic; however, not many models predict continuous centesimal reading ability. Since our predicted result is a continuous variable, thus we can also categorize the learners according to these continuous results. In the reading ability testing, 4.9% is an acceptable error range, because this error would not affect the reading ability level that an online learner belongs to. Also, compared to the other studies, our test accuracy is higher than average. (3) Objective: Some predictive models use AHP function to obtain the weights of indicators, together with the use of surveys or interviews. However, these are subjective data that can sometimes make the results invalid. Our predictive model is built on and examined through empirical eye-tracking data. These three advantages make this model stand out.

### 7.3. Implications and Future Research

This study contributes the following: first, sensitive eye-tracking indicators are found to identify online learners’ Chinese reading abilities. There are crucial differences between the Chinese and English reading processes, because the former is hieroglyphic and the latter is alphabetic. Previous studies mainly focused on alphabetic systems, thus this paper makes an important compliment to the study of eye-tracking indicators in Chinese reading.

Second, this study has built up a computational model for easier and more precise modeling of online learners’ reading ability. The model is able to predict learners’ reading ability with an average error of 4.9 points out of 100, which is an easier and more accurate way to rate learners’ reading ability. With this model, the distance education system will have the capability to identify learners’ reading ability according to their eye movement and university entrance exam scores. Online instructors can then guide learners to different kinds of learning materials according to their aptitudes. The model can be embedded in online learning systems and might also be suitable for mobile learning when learners use mobile phones with digital cameras [45,46]. 

Further research could be considered in the following directions: First, the MTJL model proposed in this study is a multi-feature regularization machine learning mechanism based on Low-rank Constraint. It is proposed based on our research question and aiming at detecting online learners’ reading ability based on their eye movements. It is quite different from other main-stream machine learning approaches (e.g., SVMs or CNNs). SVM could be applied for assessing learners’ reading ability if each of them only participated in one trial. However, in eye-tracking experiment, each participate would have to complete multiple trials, thus SVM cannot be applied directly, and need further improvement. With regard to CNN, which is a deep learning approach and usually need bigger data for training, thus it is not considered in the first place of this paper. However, it might be a good direction for adding CNN technique to improve our current model in the future. 

Second, in this paper, we studied eye-tracking indicators for identifying learners’ reading ability, while we are also interested in eye-tracking indicators that are sensitive to recognizing learners’ cognitive and emotional status. The growing body of literature contributing to identifying learners’ cognitive and emotional status according to their eye movements can add to this current research. For example, Li and Mao discovered that the stronger a visual stimulus (rated in terms of valence, no matter whether it is positive or negative), the larger the pupil size, while the blink rate decreases when the value of the stimulus is strengthened, whether it is positive or negative [47]. In addition, Mello et al. used a commercial eye tracker to monitor a student’s gaze patterns and identify when s/he is bored, disengaged, or zoning out [48]. With this information, tutors could then attempt to re-engage students with dialog moves that direct students to re-orient their attentional patterns. Is our model also able to identify learners’ fatigue and concentration status? This is another question that is worth exploring.

Third, this model can also be extended to compile indicators of facial expression and eye movements. Since current techniques for analyzing learners’ ability and status are not sufficiently developed, the identification rate is not high, thus the combined identification using both facial expression and eye movement data might be more effective. However, there may be a paradox when taking multiple sets of indicators into account during the identification process. In this machine-learning computational model, the computer will deal with the paradoxes directly, making the identification results more stable and reliable.

Fourth, this model might also be suitable for diagnosing dyslexia. Although this paper is mainly targeted at normal online learners with various reading abilities, it might also be suitable for diagnosing learners with dyslexia. This is because if this model can detect normal learners’ reading ability, then it should be even easier to detect those with dyslexia. We believe that this study provides new tools for diagnosing dyslexia for future studies, as well as measuring reading ability in different languages.

## Figures and Tables

**Table 1:** Sensitive eye-tracking indicators.

Indicator Families	Indicators	Scales	F	Sig.
**Pupil**	Pupil diameter	Pixels	2544.09	0
**Blink**	Blink count	Number of counts	156.92	0
	Blink rate	Number of blinks per second	133.89	0
**Fixation**	Fixation count	Number of fixations	15.64	0
	Fixation rate	Number of fixations per second	560.07	0
	Fixation duration	Milliseconds (ms)	18.12	0
	Fixation position X	Pixels	14.74	0
	Fixation position Y	Pixels	0.15	0.83
**Saccade**	Saccade count	Number of times	50.76	0
	Saccade rate	Number of saccades per second	4.28	0.04
	Saccade duration	Milliseconds (ms)	127.73	0
	Saccade amplitude	Degrees of visual angle	34.44	0
**Regression**	Regression count	Number of regressions	742.83	0
	Regression rate	Number of regressions per second	905.39	0
	Regression length	Pixels	1.71	0.19

**Table 2:** Comparison of assessment accuracy.

Reference	Assessment Purpose	Participants	Assessment Approach	Assessment Accuracy
Tamboer et al. [40]	Discriminate dyslexia	First-year university students	Questionnaires with 201 items and 10 reading tests with 242 items	Correct classification rate = 89.5%
Top et al. [41]	Discriminate dyslexia	First-year university students	Reading tests with three sets of predictors that extracted from the initial 27 indicators.	Correct classification rate = 90.9%
Wen [42]	Assess reading ability	Primary school students	Reading ability measurement scales	Average *α* coefficient = 0.81; composite absolute error = 5.76%; Generalizability coeffcient = 0.81; index of dependability = 0.80; criterion-related validity = 0.71.
Chan et al. [43]	Discriminate dyslexia	Primary school students	A 65-item checklist of student reading-related behavioural characteristics	Correct classification rate = 79%; kappa coefficient = 0.56
Tian [44]	Assess reading ability	Primary school students	Reading test with four-perspective structure	Test reliability Cronbach’s α= 0.85; criterion-related validity = 0.883
Our predictive model	Assess reading ability	First-year university students	Eye-tracking experiment for about 20 min	Average error = 4.91%; criterion-related validity = 0.78

## References

[1] Grubb M. (2011). Predictors of High School Student Success in Online Courses. Ph.D. Thesis.

[2] Rayner K. (2009). Eye movements and attention in reading, scene perception, and visual search. Q. J. Exp. Psychol..

[3] Chang W., Cha H., Im C. (2016). Removing the Interdependency between Horizontal and Vertical Eye-Movement Components in Electrooculograms. Sensors.

[4] Frutos-Pascual M., Garcia-Zapirain B. (2015). Assessing Visual Attention Using Eye Tracking Sensors in Intelligent Cognitive Therapies Based on Serious Games. Sensors.

[5] Hyönä J. (2010). The use of eye movements in the study of multimedia learning. Learn. Instr..

[6] Zhan Z.H. (2013). An emotional and cognitive recognition model for distance learners based on intelligent agent-the coupling of eye tracking and expression recognition techniques. Mod. Dist. Educ. Res..

[7] Zhan Z.H., Liang T., Ma Z.C. (2014). Distance learning supporting services based on virtual assistant and its technical difficulties. Mod. Dist. Educ. Res..

[8] Cassin B., Solomon S. (1990). Dictionary of Eye Terminology.

[9] Heo H., Lee W., Shin K., Park K. (2014). Quantitative Measurement of Eyestrain on 3D Stereoscopic Display Considering the Eye Foveation Model and Edge Information. Sensors.

[10] Bang J., Heo H., Choi J., Park K. (2014). Assessment of Eye Fatigue Caused by 3D Displays Based on Multimodal Measurements. Sensors.

[11] Van Gerven P.W.M., Paas F.G.W.C., Van Merriënboer J.J.G., Schmidt H.G. (2002). Cognitive load theory and aging: Effects of worked examples on training efficiency. Learn. Instr..

[12] Montés-Micó R., Alió J.L., Charman W.N. (2005). Dynamic changes in the tear film in dry eyes. Invest. Ophthalmol. Vis. Sci..

[13] Wolkoff P., Nøjgaard J.K., Troiano P., Piccoli B. (2005). Eye complaints in the office environment: Precorneal tear film integrity influenced by eye blinking efficiency. Occup. Environ. Med..

[14] Tsai Y.F., Viirre E., Strychacz C., Chase B., Jung T.P. (2007). Task performance and eye activity: Predicting behavior relating to cognitive workload. Aviat. Space Environ. Med..

[15] Holmqvist K., Nyström M., Andersson R., Dewhurst R., Jarodzka H., Van de Weijer J. (2011). Eye Tracking: A Comprehensive Guide to Methods and Measures.

[16] Jacob R.J.K., Karn K.S., Hyona J., Radach R., Deubel H. (2003). Commentary on Section 4—Eye Tracking in Human-Computer Interaction and Usability Research: Ready to Deliver the Promises. The Mind’s Eye: Cognitive and Applied Aspects of Eye Movement Research.

[17] Steichen B., Carenini G., Conati C. User-adaptive information visualization: Using eye gaze data to infer visualization tasks and user cognitive abilities. Proceedings of the 2013 International Conference on Intelligent User Interfaces.

[18] Nakayama M., Takahashi K., Shimizu Y. The act of task difficulty and eye-movement frequency for the oculo-motor indices. Proceedings of the 2002 Symposium on Eye-Tracking Research & Applications.

[19] Benedetto S., Carbone A., Drai-Zerbib V., Pedrotti M., Baccino T. (2014). Effects of luminance and illuminance on visual fatigue and arousal during digital reading. Comput. Hum. Behav..

[20] Van Orden K.F., Jung T.P., Makeig S. (2000). Combined eye activity measures accurately estimate changes in sustained visual task performance. Biol. Psychol..

[21] Volkmann F.C. (1986). Human visual suppression. Vis. Res..

[22] Rayner K. (1998). Eye movements in reading and information processing: 20 years of research. Psychol. Bull..

[23] Underwood G., Foulsham T., Humphrey K. (2009). Saliency and scan patterns in the inspection of real-world scenes: Eye movements during encoding and recognition. Vis. Cognit..

[24] Beymer D., Russell D.M., Orton P.Z. (2005). Wide vs. Narrow Paragraphs: An Eye Tracking Analysis. Human-Computer Interaction-Interact.

[25] Zhang L., Ren J., Xu L., Qiu X.J., Jonas J.B. (2013). Visual comfort and fatigue when watching three-dimensional displays as measured by eye movement analysis. Br. J. Opht..

[26] Kang M.J., Hsu M., Krajbich I.M., Loewenstein G., McClure S.M., Wang J.T.Y., Camerer C.F. (2009). The wick in the candle of learning epistemic curiosity activates reward circuitry and enhances memory. Psychol. Sci..

[27] Zekveld A.A., Heslenfeld D.J., Johnsrude I.S., Versfeld N.J., Kramer S.E. (2014). The eye as a window to the listening brain: Neural correlates of pupil size as a measure of cognitive listening load. NeuroImage.

[28] Yan G., Tian H., Bai X., Rayner K. (2006). The effect of word and character frequency on the eye movements of Chinese readers. Br. J. Psychol..

[29] Ehmke C., Wilson C. Identifying web usability problems from eye tracking data. Proceedings of the 21st British HCI Group Annual Conference on People and Computers.

[30] Reingold E.M., Charness N., Pomplun M., Stampe D.M. (2001). Visual span in expert chess players: Evidence from eye movements. Psychol. Sci..

[31] Tatler B.W., Gilchrist I.D., Land M.F. (2005). Visual memory for objects in natural scenes: From fixations to object files. Q. J. Exp. Psychol. Sect. A.

[32] Reingold E.M., Charness N., Underwood G. (2005). Perception in chess: Evidence from eye movements. Cognitive Processes in Eye Guidance.

[33] Savelsbergh G.J., Williams A.M., Kamp J.V.D., Ward P. (2002). Visual search, anticipation and expertise in soccer goalkeepers. J. Sports Sci..

[34] Van Orden K.F., Limbert W., Makeig S., Jung T.P. (2001). Eye activity correlates of workload during a visuo spatial memory task. Hum. Factors.

[35] Vuori T., Olkkonen M., Pölönen M., Siren A., Häkkinen J. Can eye movements be quantitatively applied to image quality studies?. Proceedings of the Third Nordic Conference on Human-computer Interaction.

[36] Tatler B.W., Baddeley R.J., Vincent B.T. (2006). The long and the short of it: Spatial statistics at fixation vary with saccade amplitude and task. Vis. Res..

[37] Kuperman V., Van Dyke J.A. (2011). Effects of individual differences in verbal skills on eye-movement patterns during sentence reading. J. Mem. Lang..

[38] Rayner K., Ardoin S.P., Binder K.S. (2013). Children’s eye movements in reading: A commentary. Sch. Psychol. Rev..

[39] Kasarskis P., Stehwien J., Hickox J., Aretz A., Wickens C. Comparison of expert and novice scan behaviors during VFR flight. Proceedings of the 11th International Symposium on Aviation Psychology.

[40] Tamboer P., Vorst H.C., Oort F.J. (2014). Identifying dyslexia in adults: An iterative method using the predictive value of item scores and self-report questions. Ann. Dyslexia.

[41] Tops W., Callens M., Lammertyn J., Van Hees V., Brysbaert M. (2012). Identifying students with dyslexia in higher education. Ann. Dyslexia.

[42] Wen H. (2005). Primary School students’ Chinese Reading Ability Scales: Development, Validity, and Reliability. Master’s Thesis.

[43] Chan D.W., Ho C.S., Tsang S.M., Lee S.H., Chung K.K. (2004). Screening for Chinese children with dyslexia in Hong Kong: The use of the teachers’ behaviour checklist. Educ. Psychol..

[44] Tian X. (2003). Research of Primary School Students’ Reading Ability. Master’s Thesis.

[45] Lopez-Basterretxea A., Mendez-Zorrilla A., Garcia-Zapirain B. (2015). Eye/Head Tracking Technology to Improve HCI with iPad Applications. Sensors.

[46] Paravati G., Gatteschi V. (2015). Human-Computer Interaction in Smart Environments. Sensors.

[47] Li Z., Mao X. (2012). Emotional eye movement generation based on Geneva Emotion Wheel for virtual agents. J. Vis. Lang. Comput..

[48] D’Mello S., Olney A., Williams C., Hays P. (2012). Gaze tutor: A gaze-reactive intelligent tutoring system. Int. J. Hum. Comput. Stud..

